# Ranolazine as a First-Choice Anti-anginal Medication for Patients With Coronary Artery Ectasia: A Case Series

**DOI:** 10.7759/cureus.52747

**Published:** 2024-01-22

**Authors:** Juwayria A Ahmed, Khudheeja A Ahmed, Mohammed Habeeb Ahmed

**Affiliations:** 1 Department of Research, KAAJ Healthcare, San Jose, USA; 2 Cardiology, KAAJ Healthcare, San Jose, USA

**Keywords:** coronary slow flow phenomenon, angina pectoris, ranolazine, aneurysmal coronary artery disease, coronary artery ectasia (cae)

## Abstract

Coronary artery ectasia (CAE) is characterized by the abnormal dilation of coronary arteries, resulting in disturbed or slow blood flow, which causes angina pectoris-the most prevalent symptom of CAE. To date, there is no consensus on the therapeutic management of CAE due to its rarity and the scarcity of research. We present a case series of five patients with different ethnicities, including both men and women, whose CAE was successfully managed by the administration of ranolazine. All five patients were found to have CAE by coronary angiography, which was also associated with slow blood flow. Clinically, the patients had accelerating angina. They were prescribed an initial dose of 500 mg of ranolazine twice daily, which led to the resolution of their anginal symptoms. They have been clinically and hemodynamically stable for the last several years. In light of these results, we propose that ranolazine be considered as a first-choice anti-anginal medication for patients with CAE.

## Introduction

Coronary artery ectasia (CAE), also known as aneurysmal coronary artery disease (CAD), is characterized by the dilation of a segment of the coronary artery lumen to a diameter at least 1.5 times greater than that of the adjacent normal coronary artery [1, 2]. CAE can present as either diffuse, affecting the entire length of the coronary artery, or as localized, affecting a segment of the artery [1]. The term 'coronary aneurysm' is more commonly used to describe localized and saccular-type ectatic segments, while 'ectasia' is more commonly used for fusiform diffuse ectasia [1]. The most frequent locations for CAE are the right coronary artery (68%), the proximal left anterior descending artery (60%), and the left circumflex artery (50%) [2]. CAE is classified into four types in decreasing order of severity: Type 1 involves diffuse ectasia in two or three vessels; Type 2, diffuse ectasia in one vessel and localized disease in another; Type 3, diffuse ectasia in only one vessel; and Type 4, localized or segmental ectasia [3]. CAE is an uncommon phenomenon, with an incidence of between 0.3% and 4.9% [2]. Coronary angiography is the gold standard for diagnosing CAE [2]. Aneurysmal segments cause disturbances in blood flow filling and washout, resulting in slow, turbulent, or stagnant blood flow, which is visualized by the flow of contrast used in the angiography [1]. The most prevalent symptom of CAE is angina pectoris, which can be caused by slow flow in the coronary arteries even in the absence of associated coronary artery stenosis [4].

CAE is caused by atherosclerosis in 50% of cases and often coexists with CAD in a significant majority of patients [1]. It has also been associated with connective tissue disorders such as scleroderma, Ehlers-Danlos syndrome, syphilitic aortitis, arteritis, Marfan syndrome, polyarteritis nodosa, Takayasu disease, systemic lupus erythematosus, and Kawasaki disease [1, 2], with Kawasaki disease being the most common cause in children and young adults [5]. Additionally, 20-30% of cases are congenital [1]. Iatrogenic causes, including interventions like percutaneous transluminal coronary angioplasty (PTCA), stenting, and directional coronary atherectomy, can also lead to CAE [2]. CAE is rarely genetic [2]. Notably, recent studies suggest an association between CAE and aneurysms in multiple vascular territories, with CAE being more prevalent in patients with aneurysms affecting the abdominal and ascending aorta, popliteal arteries, and the pulmonary artery [1].

There is no clear consensus on the medical management of CAE [1, 2]. Due to the blood flow disturbances from ectatic segments, chronic anticoagulants have been proposed as a therapeutic option for CAE patients, but this treatment needs to be tested in prospective studies before it can be recommended [1]. Antiplatelet therapy, along with the addition of adenosine diphosphate inhibitors, is considered in cases of isolated CAE, but their effectiveness has not yet been confirmed by prospective studies [1]. Medications with vasodilating properties have also been suggested, but there are currently no widely tested vasoactive medications that can be recommended for CAE patients [1]. Notably, nitrates are not recommended for patients with CAE as they cause further coronary epicardial dilation, which can exacerbate myocardial ischemia [1]. Heparin infusion and fibrinolysis have been successfully used for recanalization on occasion [1]. Additionally, a study by Sudhir K et al. found that CAE is six times more common in patients with a history of familial hypercholesterolemia, suggesting that lipid-lowering agents may be a potential treatment for CAE [6].

When CAE coexists with CAD, patients are usually treated for CAD only [1]. Since CAE often coexists with obstructive coronary lesions, posing a subsequent risk of myocardial infarction, aspirin has been suggested as treatment for all patients with CAE [1]. For patients whose symptoms of significant ischemia persist despite medical management, percutaneous/surgical revascularization may be performed to restore normal myocardial perfusion [1]. Ochiai et al. reported favorable long-term outcomes with balloon angioplasty in lesions near coronary aneurysms [1]. However, when performing percutaneous/surgical revascularization in ectatic arteries, it is essential to pay special attention to ensuring sufficient stent sizing and expansion, as well as wall stabilization. For significant CAD alongside CAE, coronary artery bypass grafting may be utilized for patients with recurring complications, generally yielding positive results. However, surgery is generally avoided in most cases [1, 2].

## Case presentation

Case 1

A 75-year-old Caucasian male presented to the cardiologist’s office with symptoms of worsening angina. His stress test was positive for reversible ischemia involving the inferior wall. He had a history of hypertension, dyslipidemia, left ventricular hypertrophy (LVH), arrhythmias, cardiomegaly, and transient ischemic attack (TIA). The patient was taking the following medications at the time: aspirin 81 mg daily, amlodipine 10 mg daily, clopidogrel 75 mg daily, simvastatin 40 mg, losartan 100 mg daily, and carvedilol 12.5 mg twice daily. Cardiac catheterization showed the following findings: The left anterior descending artery (LAD) was aneurysmal and ectatic in its proximal portion with slow flow visualized. There were luminal irregularities in multiple areas. The circumflex coronary artery was aneurysmal and ectatic in its proximal and mid portions. The right coronary artery was a large, dominant vessel with aneurysmal, ectatic portions and a ruptured plaque proximally, along with diffuse disease throughout its course. The ascending aorta was also dilated, measuring 4.5 cm.

Case 2

**Figure 1:**
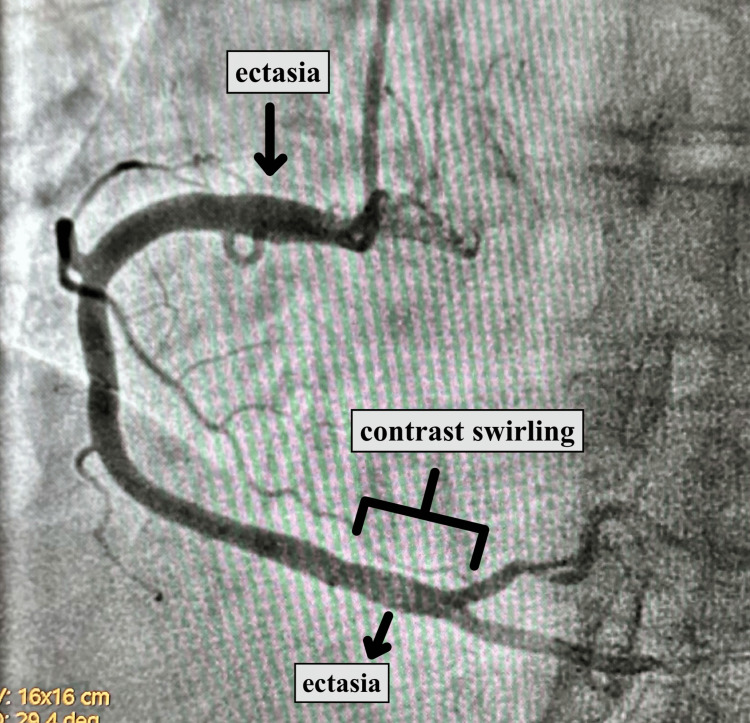
Angiography reveals the presence of ectasia in the right coronary artery, along with a noticeable slow flow phenomenon observed in the distal right coronary artery where the contrast swirls before its bifurcation.

A 52-year-old Filipina female presented to the cardiologist’s office with complaints of episodes of chest pain, occurring intermittently for the past few weeks. The pain was substernal in location and radiated to the left arm. The episodes occurred with no aggravating factors and were associated with palpitations. Workup, including a cardiolyte stress test, showed no evidence of adenosine-induced ischemia and an ejection fraction (EF) of 72%. The patient had a medical history of hypertension, dyslipidemia, bradycardia, LVH, and diastolic dysfunction. At the time, the patient was taking the following medications: Simvastatin 10 mg, aspirin 81 mg daily, losartan 100 mg daily, and metoprolol 50 mg daily. Cardiac catheterization showed that the LAD artery was significantly dilated proximally with no obstructive disease. The circumflex coronary artery was also dilated proximally. The right coronary artery was dilated both proximally and distally and was almost aneurysmal. In the distal area just prior to the bifurcation of the posterior descending artery (PDA) and posterolateral vein (PLV), a slow flow phenomenon was observed in the distal right coronary artery, with the contrast swirling around, as seen in Figure 1.

Case 3

**Figure 2:**
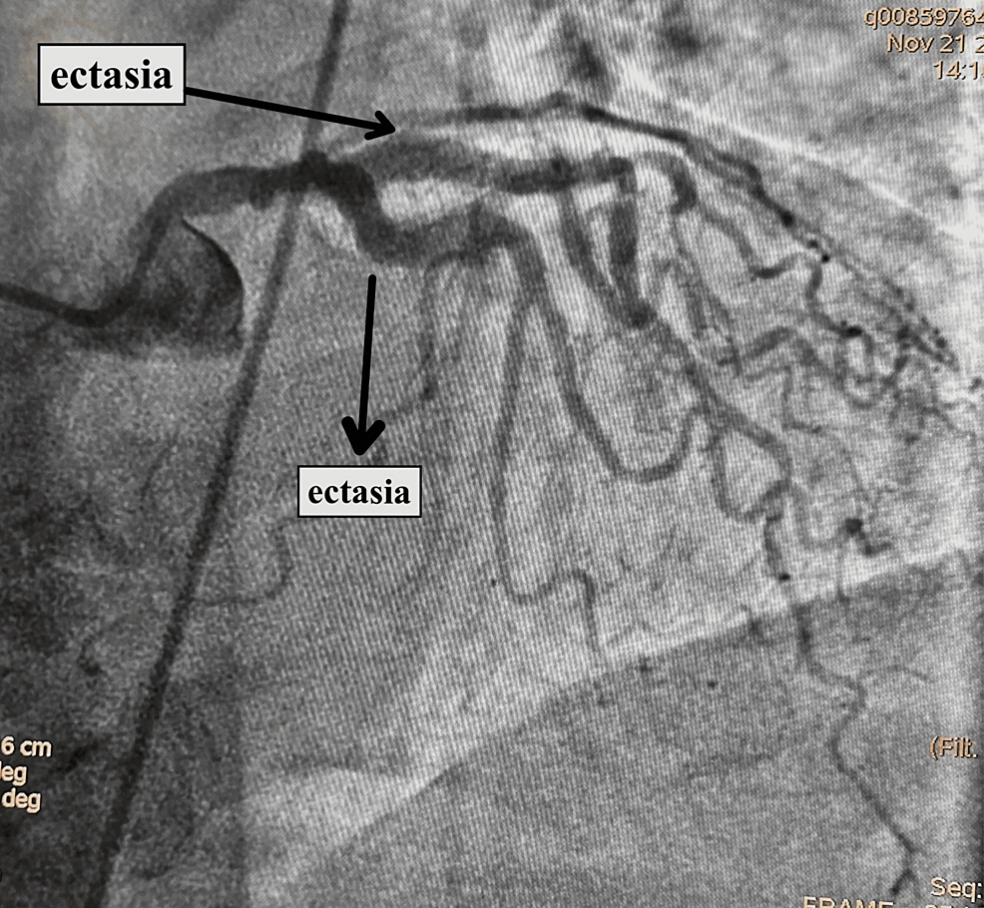
Angiography showing ectasia in the LAD as well as the circumflex coronary artery. LAD: Left anterior descending artery.

A 64-year-old Mexican male presented to the cardiologist’s office with complaints of episodes of palpitations, occurring intermittently for the past few weeks. The episodes were associated with dizziness and fatigue. He had a history of arrhythmias with tachy-brady syndrome. Additionally, he had a history of hypertension, dyslipidemia, LVH, cardiomegaly, cardiomyopathy, diabetes mellitus, and chronic alcoholism. He also had a history of multiple falls. At the time of the visit, the patient was taking the following medications: Eliquis 5 mg twice daily, aspirin 81 mg daily, metoprolol 25 mg daily, and furosemide 20 mg daily. An adenosine cardiolyte stress test done earlier showed no evidence of stress-induced ischemia, an ejection fraction (EF) of 40-45%, and mild inferior wall hypokinesis. Cardiac catheterization revealed that the left main was tapered distally in the range of 30% to 40%, dividing the LAD artery and circumflex coronary artery. The LAD showed significant calcification and was aneurysmal, as seen in Figure 2, with an ostial 30% to 40% stenosis and mid 50% to 60% stenosis. The first diagonal artery was a large vessel with an ostial/proximal 40% to 50% stenosis. The circumflex coronary artery had an ostial 40% to 50% stenosis and luminal irregularities distally. The right coronary artery, again, was a large aneurysmal vessel with proximal 20% to 30% stenosis and mid 30% to 40% stenosis. Ventriculography showed an EF calculated at 32%; however, due to atrial fibrillation and PVCs, EF may not be accurate.

Case 4

A 70-year-old Mexican female presented to the cardiologist’s office with complaints of shortness of breath, progressively worsening over the past few weeks, as well as episodes of palpitations associated with dizziness and fatigue, occurring intermittently during the same period. A recent adenosine cardiolyte stress test showed a small, mild, partially reversible anterior wall defect suggestive of adenosine-induced ischemia, comprising 5% of the left ventricular myocardial volume, with an EF of 62%. The patient had a history of hypertension, dyslipidemia, LVH, cardiomegaly, and arrhythmias. Holter monitoring had shown runs of supraventricular tachycardia (SVT). At the time of the visit, the patient was taking the following medications: levothyroxine 80 mg, aspirin 81 mg, losartan 50 mg, metronidazole 500 mg, Toprol 25 mg, and atorvastatin 10 mg. Cardiac catheterization revealed that the LAD artery was aneurysmal in its proximal and midportion and extremely narrowed after the large diagonal artery. The circumflex coronary artery was aneurysmal in its proximal and midportion. The vessel tapered in the midportion in the range of 55% to 60%, and after the first obtuse marginal artery, the tapering was in the range of 35% to 40%. The right coronary artery was a large dominant vessel with a significant aneurysmal portion in the proximal and midportion. The ostial proximal area had a narrowing of about 60% to 70%, and in the midportion, there was another 50% to 60% narrowing.

Case 5

**Figure 3:**
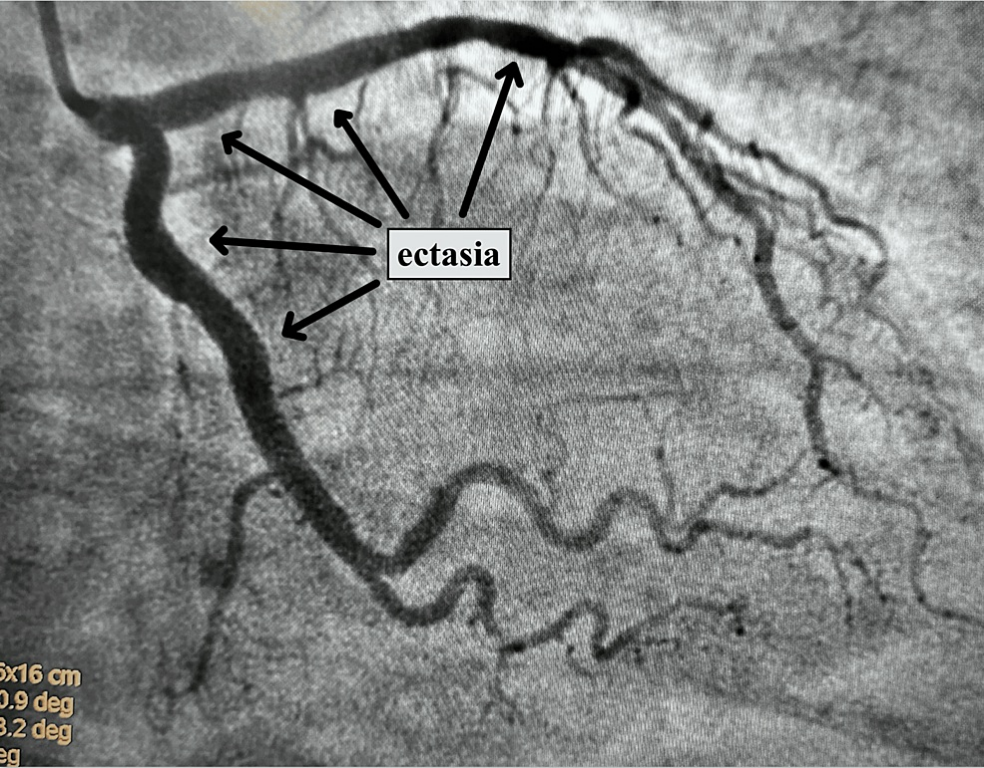
Angiography showing ectasia in the LAD as well as the circumflex coronary artery. LAD: Left anterior descending artery.

A 76-year-old Portuguese female presented to the cardiologist’s office with complaints of episodes of palpitations, occurring intermittently over the past few weeks and associated with dizziness and fatigue. She was also experiencing episodes of shortness of breath, progressively worsening during the same period. Previous workup included a recent adenosine cardiolyte stress test, which revealed a medium-sized, predominantly fixed perfusion defect involving the inferior wall with a small portion demonstrating partial reversibility. Her EF was measured at 60-65%. The patient had a history of hypertension, dyslipidemia, arrhythmias, LVH, diastolic dysfunction, and asthma. At the time of the visit, she was taking the following medications: atorvastatin 40 mg, metoprolol 50 mg, losartan potassium 25 mg, amlodipine 10 mg, hydrochlorothiazide 25 mg, aspirin 81 mg, and ozempic 25 mg. Cardiac catheterization revealed proximal dilation of the LAD artery with tapering in the mid portion and 40% to 50% narrowing. The circumflex coronary artery also exhibited dilatation in its proximal and mid portions, as shown in Figure 3. The right coronary artery displayed aneurysmal portions with no obstructive disease. Additionally, a slow flow phenomenon was observed in all the vessels.

## Discussion

CAE involves the dilation of the coronary artery lumen and is frequently associated with CAD [2]. Angina pectoris, a common symptom of CAE, is induced by the slow flow phenomenon linked to CAE, even in the absence of concurrent obstructive CAD [2]. Due to the rarity of this condition and limited research in the field, there is currently no established therapeutic approach for its management [2].

A comprehensive literature review conducted in 2021 by Khedr A et al., focusing on various pharmacological approaches used for CAE, discovered significant elevations in mean platelet volume in CAE patients. As a result, antiplatelet therapy, such as aspirin, may have a role in managing CAE [7]. Anticoagulants, such as warfarin, were also found to be effective in preventing thrombus formation in some studies. However, use of anticoagulants requires case-by-case attention, considering the presence of concomitant obstructive CAD and the patient's risk of bleeding [7]. Given that atherosclerosis is the primary cause of CAE and its association with familial hypercholesterolemia, statins are recommended for all CAE patients as a primary preventive measure [6, 7]. Angiotensin-converting enzyme (ACE) inhibitors are considered potential treatment options, especially for hypertensive patients, as they may prevent the progression of coronary dilation by reducing intramural pressure [7]. Beta-blockers could also be considered due to their antihypertensive and anti-ischemic effects, although they may increase the risk of coronary vasospasm [7]. Calcium channel blockers, with their anti-spasm effects, can be used in conjunction with aspirin and warfarin to manage CAE. They may also be beneficial in countering the slow flow phenomenon associated with CAE [7]. However, nitrates, commonly used vasodilators for ischemic heart disease, are generally contraindicated in CAE as they may exacerbate symptoms [7]. Trimetazidine, another antianginal agent, has demonstrated anti-ischemic effects without significantly affecting heart rate or blood pressure and may be employed to manage anginal symptoms in CAE patients [7]. Ranolazine hydrochloride is a piperazine derivative primarily employed as a second-line antianginal medication in stable CAD [8]. It functions by blocking late sodium channels to prevent the downstream increase in cytosolic calcium, ultimately reducing left ventricular wall tension and enhancing coronary blood flow to alleviate angina [8]. Beyond its role in angina relief, ranolazine has shown promise in managing arrhythmias, notably atrial fibrillation, as well as addressing conditions such as pulmonary arterial hypertension, diastolic dysfunction, and chemotherapy-induced cardiotoxicity [8]. Several studies have even suggested the potential utility of ranolazine in the treatment of non-cardiovascular issues, including diabetes mellitus and neurological conditions characterized by myotonia [8].

In this case series, we present five patients with CAE whose angina symptoms were effectively managed with ranolazine. All five of these patients initially presented with angina, and their coronary angiography revealed the presence of CAE along with the slow flow phenomenon, as clearly demonstrated by the swirling contrast within these vessels. The initial prescribed dose was 500 mg twice daily, although some patients benefited from an increased dosage of 1000 mg twice daily. Upon administering ranolazine to these patients, their angina symptoms resolved, and they have remained clinically stable and symptom-free for an extended period. For instance, the patient mentioned in Case 1 has been angina-free for the past eight years while taking ranolazine. Follow-up stress tests for all patients have yielded negative results for reversible ischemia. Based on the significant success observed in these cases, we recommend considering ranolazine as the first-choice anti-anginal medication for patients diagnosed with CAE. Despite being identified over 50 years ago, the therapeutic management of CAE remains a topic of ongoing debate, with no known consensus to date [7]. The low incidence ranging from 0.3% to 4.9% of CAE has resulted in insufficient data for conducting large randomized controlled trials and prospective studies to confirm the effectiveness of various treatments [2]. This scarcity of research poses challenges in effectively managing CAE. This case series contributes to the existing literature by demonstrating the successful management of anginal symptoms associated with CAE using ranolazine. Further research is recommended to address this gap in the literature on the effectiveness of each proposed CAE treatment. 

## Conclusions

CAE is the dilation of the coronary artery lumen and is associated with disturbed blood flow, leading to symptoms of angina. Due to the rarity of the condition and scarcity of research in the area, there is no clear therapeutic approach for management of CAE. We present a case series featuring five patients with CAE and whose symptoms of angina were successfully managed by ranolazine. Based on the effectiveness of ranolazine for these patients, we propose that ranolazine be considered as a first-choice anti-anginal medication for patients with CAE. Further research with multi-center randomized control trials is needed.
